# Risk factors for progression to castration-resistant prostate cancer in metastatic prostate cancer patients

**DOI:** 10.7150/jca.30731

**Published:** 2019-09-07

**Authors:** Ting-Ting Lin, Ye-Hui Chen, Yu-Peng Wu, Shao-Zhan Chen, Xiao-Dong Li, Yun-Zhi Lin, Shao-Hao Chen, Qing-Shui Zheng, Yong Wei, Ning Xu, Xue-Yi Xue

**Affiliations:** Departments of Urology, the First Affiliated Hospital of Fujian Medical University, 20 Chazhong Road, Fuzhou 350005, China

**Keywords:** metastatic prostate cancer, androgen deprivation therapy, castration-resistant prostate cancer, progression-free survival, risk factors

## Abstract

**Purpose**: To investigate the risk factors for progression to castration-resistant prostate cancer (CRPC) in metastatic prostate cancer (mPCa) patients who underwent androgen deprivation therapy (ADT).

**Methods**: We analyzed 216 patients with mPCa who underwent ADT between January 2006 and December 2015 at the First Affiliated Hospital of Fujian Medical University. Univariate and multivariate Cox regression analysis were used to explore the risk factors for progression to CRPC. Kaplan-Meier analysis and log-rank test were used to evaluate the difference in progression-free survival (PFS).

**Results**: A total of 121 (56.0%) patients who underwent ADT showed progression to CRPC. Multivariate Cox regression analysis demonstrated that Gleason grade group, prostate-specific antigen nadir (nPSA), and time to PSA nadir (TTN) were risk factors for progression to CRPC in mPCa patients. Kaplan-Meier analysis demonstrated that patients in Gleason grade group ≥3, nPSA >0.2 ng/ml and TTN <6 months had shorter PFS.

**Conclusion**: This study demonstrated that Gleason grade group, nPSA and TTN were risk factors for progression to CRPC. Patients with higher Gleason grade group, higher nPSA and shorter TTN have shorter PFS and higher risk of progression to CRPC after ADT.

## Introduction

Recently, prostate cancer (PCa) has become one of the most common malignant tumors in western countries 1-3. The incidence of PCa in China has increased in recent years 4. Patients with bone or visceral metastasis can be diagnosed with metastatic PCa (mPCa) 5.

Patients with mPCa usually have no opportunity to undergo radical treatment. Instead, androgen deprivation therapy (ADT) works as the first-line treatment to prevent progression to castration-resistant PCa (CRPC)^6^. However, patients who undergo ADT inevitably show progression to CRPC 7. Patients with CRPC have poor prognosis and unsatisfactory therapeutic effects.

Thus, it is important to identify the risk factors for rapid progression to CRPC in patients who initially respond to ADT. This study explored the risk factors for progression to CRPC in patients with mPCa who underwent ADT.

## Materials and Methods

### Patients and data collection

We retrospectively reviewed the clinical data of mPCa patients between January 2006 and December 2015. The regiments of patients received ADT in this study were luteinizing hormone-releasing hormone agonists (LHRH-A) accompanied with an anti-androgen until progression to CRPC or at the end of follow-up time. The inclusion criteria were as follows: patients diagnosed with pathologically confirmed PCa and have positive findings of bone or visceral metastasis. The exclusion criteria were as follows: patients with other cancer, treated with 5α-reductase inhibitors within 6 months, received chemotherapy or radiotherapy during the follow-up time, or without whole course intervention of ADT were excluded in this study. Age, body mass index, prostate-specific antigen (PSA) baseline level, Gleason score and Gleason grade group were recorded in a database, as well as the level of alkaline phosphatase, lactate dehydrogenase and hemoglobin. Gleason score was subject to pathological diagnosis by prostate biopsy. Patients were divided into 5 groups according to the Gleason grade group system devised in 2014 by the International Society of Urological Pathology 8: Grade group 1, Gleason score ≤6; Grade group 2, Gleason score 3+4=7; Grade group 3, Gleason score 4+3=7; Grade group 4, Gleason score 8; and grade group 5, Gleason score ≥9. Bone and visceral metastasis were diagnosed by bone emission computed tomography, magnetic resonance imaging of the pelvis, and computed tomography of the chest and abdomen. All patients received ADT regularly.

### Follow-up

The duration of response to ADT in each patient was determined by the detection of serum PSA levels every 3 months. PSA nadir (nPSA) and time to PSA nadir (TTN) were recorded during follow-up. nPSA was classified as ≤0.2 ng/ml or >0.2 ng/ml, and TTN as <6 months or ≥6 months, based on previously published studies 9-11. The progression to CRPC was defined as castrate serum testosterone <50 ng/dl or 1.7 nmol/l, in addition to either biochemical progression (3 consecutive rises in PSA 1 week apart, resulting in 50% increases over the nadir, with PSA >2 ng/ml), or radiological progression, based on the 2017 European Association of Urology guidelines 12, 13.

### Statistical analysis

Statistical analysis was performed using SPSS version 22.0 (SPSS, Chicago, IL, USA). Survival curves were generated using the Kaplan-Meier method. Univariate and multivariate Cox regression analyses were performed to explore the risk factors associated with progression to CRPC. P<0.05 was considered statistically significant.

## Results

A total of 635 patients were diagnosed as metastatic prostate cancer in our center during the study period. A number of 419 patients were excluded in this study. 8 patients diagnosed with other cancers, 293 patients received 5α-reductase inhibitors or chemotherapy or radiotherapy, 11 patients refused to receive ADT, and 107 patients without complete follow-up data were excluded from this study. Finally, we included 216 patients with mPCa (**Table 1**). Bone metastasis was found in 210 (97.2%) cases, axial metastasis in 192 (88.9%), limb bone metastasis in 66 (30.6%), visceral metastasis in 46 (21.3%), and lymph node metastasis in 198 (91.7%).

**Figure 1:**
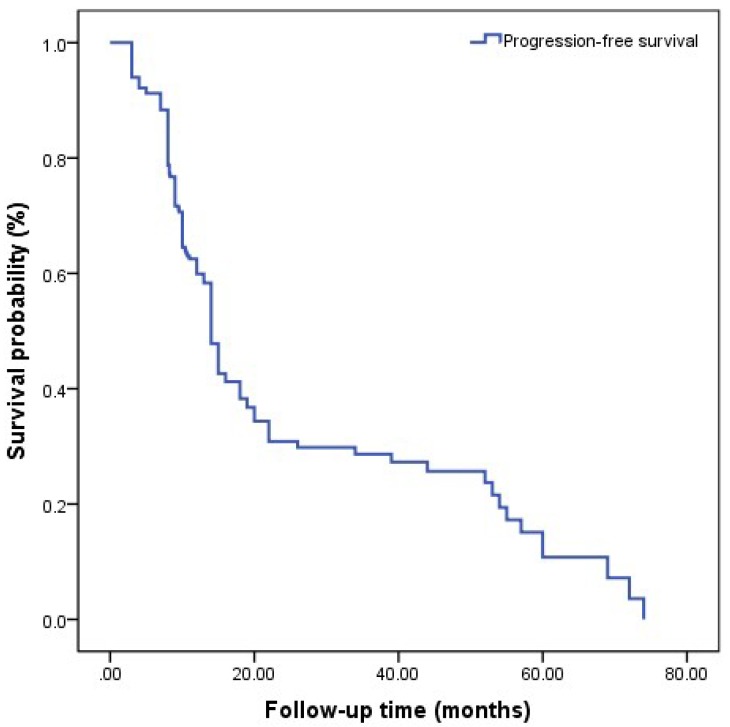
Kaplan-Meier analysis for progression-free survival in patients with advanced prostate cancer underwent androgen deprivation therapy.

Progression-free survival (PFS) is shown in (**Figure 1**). A total of 121(56.0%) patients developed to CRPC. All patients included in this study were diagnosed to have progression to CRPC by PSA changes and a total of 82 patients were diagnosed to have progression to CRPC by imaging. The median follow-up time was 14.0 (3.0-88.0) months, and the median PFS was 14.7 (3.0-74.0) months. The median nPSA was 3.23 (0-18.1) ng/ml, and median TTN was 8.10 (2-42) months.

Univariate Cox regression analysis showed that Gleason grade group, limb bone metastasis, visceral metastasis, alkaline phosphatase, PSA baseline level, nPSA, and TTN were significantly associated with progression to CRPC. Multivariate analysis revealed that patients in Gleason grade group 3 showed a 3.169-fold higher risk for progression to CRPC than those in group 1 (P=0.006). Furthermore, patients in Gleason grade group 4 showed a 4.335-fold higher risk for progression to CRPC than those in group 1 (P<0.001). Finally, patients in Gleason grade group 5 showed a 5.159-fold higher risk for progression to CRPC than those in group 1 (P<0.001). P for trend was calculated and showed that increasing Gleason grade group was significantly associated with higher risk of progression to CRPC (P for trend <0.001). Multivariate Cox regression analysis also showed that nPSA >0.2 ng/ml (hazard ratio 2.665, 95% confidence interval 1.495-4.750, P<0.001) was associated with poor PFS when compared with nPSA ≤0.2 ng/ml. However, TTN ≥6 months (hazard ratio 0.262, 95% CI 0.161-0.426) (**Tables 2 and 3**) was associated with better PFS when compared with TTN <6 months.

**Figure 2:**
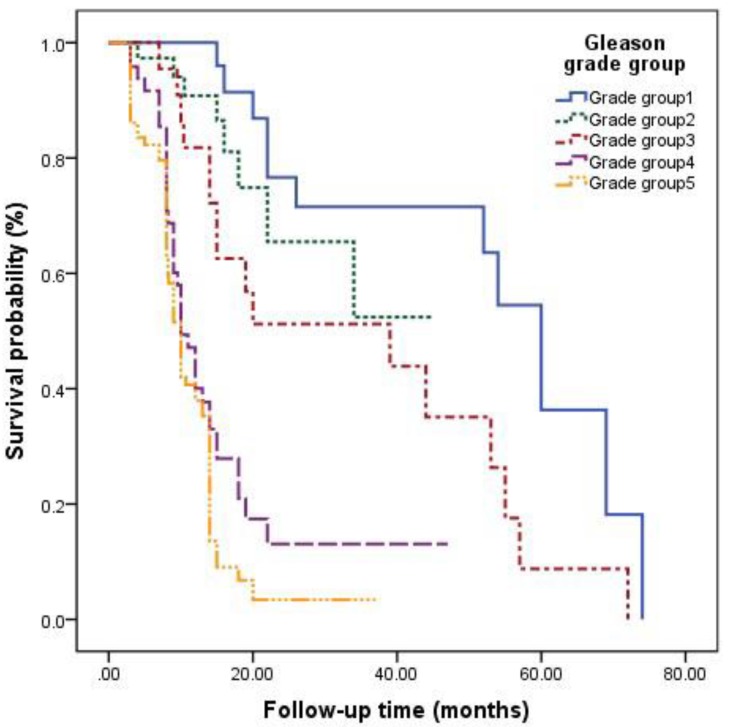
Kaplan-Meier analysis for progression-free survival in patients with advanced prostate cancer underwent androgen deprivation therapy stratified by Gleason grade group.

**Figure 3:**
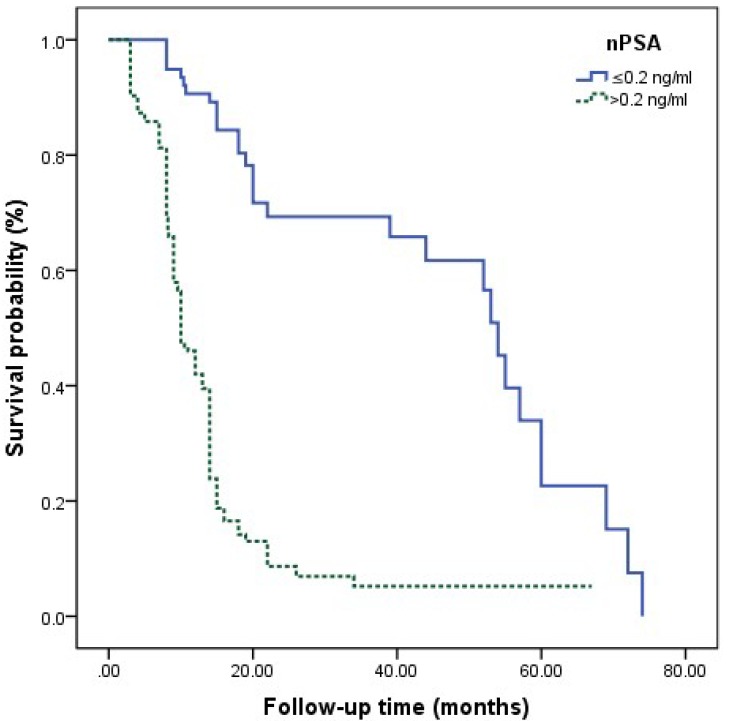
Kaplan-Meier analysis for progression-free survival in patients with advanced prostate cancer underwent androgen deprivation therapy stratified by nadir PSA.

**Figure 4:**
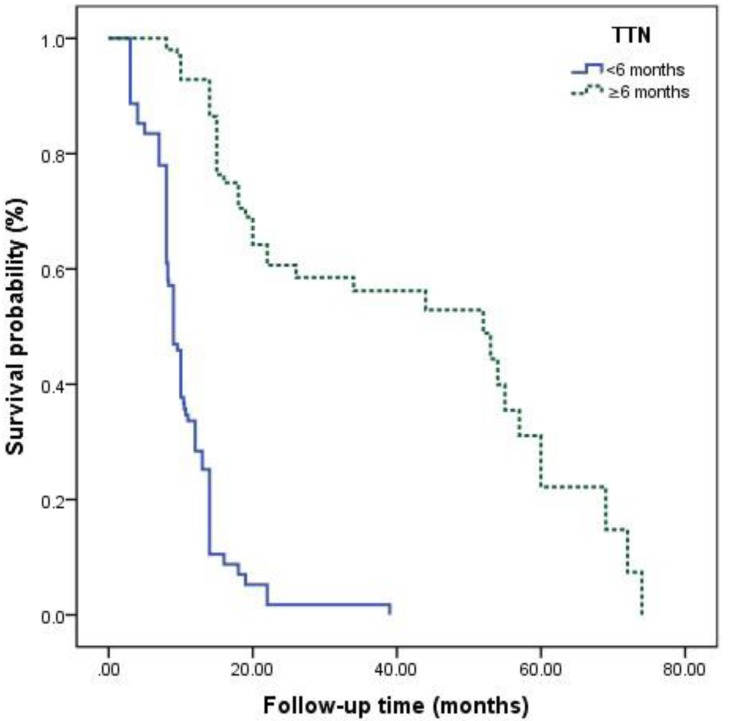
Kaplan-Meier analysis for progression-free survival in patients with advanced prostate cancer underwent androgen deprivation therapy stratified by time to nadir PSA.

The survival curves among different Gleason grade groups, nPSA, and TTN were generated (**Figures 2**-**4**). The results demonstrated that there were significant differences in PFS between patients with different Gleason grade group, nPSA and TTN (all P<0.001). PFS was worse in patients with increasing Gleason grade group, higher nPSA and shorter TTN.

## Discussion

The Gleason scoring system is a well-established predictor for staging, progression and prognosis in PCa 14. Yang et al. 15 reported that Gleason score was associated with survival of PCa patients with bone metastasis. The overall survival of patients with Gleason score ≤7 was significantly longer than in patients with score >7. Yigitbasi et al.^16^ explored the survival time of patients with mPCa who underwent ADT and found that median survival time was 33, 19 and 13 months in patients with Gleason score of 2-4, 5-7 and 8-10, respectively. In this study, we found that patients with mPCa had easy progression to CRPC if they were classified into a high Gleason grade group. We demonstrated that patients in Gleason grade groups 3, 4 and 5 showed a 3.169-, 4.335- and 5.159-fold higher risk, respectively, of progression to CRPC when compared with those in group 1. The Gleason grade group is one of the risk factors for progression to CRPC in patients with mPCa.

For PCa, PSA is a widely used serological index in diagnosis, evaluation of therapeutic effect and prediction of prognosis 17-19. Nayyar et al.^11^ reported that higher PSA baseline level was associated with poorer therapeutic effect of ADT and shorter time to progression to CRCP. However, Yamamoto et al. 20 demonstrated that patients with PSA baseline level <10 ng/ml had poorer therapeutic effect than those with ≥10 ng/ml. Some previous studies also showed that PSA baseline level cannot work as a predictor for prognosis, which was supported by the present study 21, 22. Recently, it was shown that nPSA and TTN seem to have better efficacy for prediction of prognosis than PSA baseline level has. Choueiri et al. 9 reported that TTN <6 months and nPSA >0.2 ng/ml predicted shorter overall survival in patients who had hormone-sensitive mPCa treated with ADT. A retrospective study about ADT for PCa or mPCa by Ji et al. 21 indicated that TTN ≤9 months and nPSA <0.03 ng/ml were significantly connected with an increased risk of progression to CRPC. Kuo et al. 23 reported a significant relationship between a longer time to PSA rise during the first off-treatment interval and a longer time to CRPC progression in patients treated with ADT. Besides, nPSA and TTN seem to work as predictors for prognosis in chemotherapy for CRPC 23. It is generally considered that a rapid decline of PSA indicates a higher proportion of PCa cell death and, therefore, higher survival 24. However, this is not consistent with what mentioned above. The underlying mechanisms are still unclear. It is possible that a rapid decrease of PSA might reflect down-regulation of PSA expression of hormone-sensitive PCa cells, which are regulated by androgens via the androgen receptor pathway 25. Another possibility is that rapid removal of hormone-sensitive PCa cells might induce an environment that is conducive to the growth of CRPC cells 25. Besides, a high nPSA means that many cancer cells develop into castration-resistant cells and survive ADT. Thus, more attention must be paid to patients with higher nPSA and shorter TTN for early identification of progression to CRPC.

Distant metastasis has been found in >80% of patients who died of PCa^2^. PCa usually tends to spread to axial bone rather than limb bone or viscera 26. Rigaud et al. 26 reported that patients with axial bone metastasis have better survival than those with limb bone or visceral metastases. In addition, several studies have revealed that the presence of greater bone metastasis before ADT initiation results in earlier progression to CRPC 27.

The present study had several limitations. First, this was a retrospective analysis performed at a single institution, which restricts the application and generalization of our findings. We did not evaluate other biomarkers of androgen receptors because of technological limitations. Second, the univariate analysis revealed that limb bone metastasis, visceral metastasis and level of alkaline phosphatase might predict the progression to CRPC in patients with mPCa treated with ADT. However, multivariate analysis demonstrated opposite conclusions compared with univariate analysis. Third, multivariate analysis is more reliable than univariate analysis when considering the interaction among confounding factors. For instance, when compared to the studies of Rigaud 26 and Howard 27, patients included in this study have higher Gleason grade group, which possibly reduces the effects of bone metastasis and visceral metastasis towards the results. Finally, bias resulting from small sample size, relatively short follow-up time, and patient imposed selection are usually inevitable. Therefore, further study should be conducted to validate these conclusions.

In conclusion, this study demonstrated that higher Gleason grade group, higher nPSA, and shorter TTN were associated with higher risk of progression to CRPC in patients with mPCa. Further studies are needed to confirm these conclusions.

## Clinical Practice Points

Patients with CRPC have poor prognosis and unsatisfactory therapeutic effects. This study demonstrated that Gleason grade group, nPSA and TTN were risk factors for progression to CRPC. Patients with higher Gleason grade group, higher nPSA and shorter TTN have shorter PFS and higher risk of progression to CRPC after ADT. Further studies are needed to confirm these conclusions.

## Figures and Tables

**Table 1:** Characteristics of patients with metastatic prostate cancer underwent androgen deprivation.

			X±S/No	
**Age (year)**			70.86±8.15 (47-86)	
**BMI (Kg/m^2^)**			22.29±3.15 (16.10-30.40)	
**Gleason score**			8.06±1.22 (4-10)	
**Gleason grade group (%)**				
Grade group1	≤ 6 points		29 (13.4)	
Grade group2	3+4 points		37 (17.1)	
Grade group3	4+3 points		23 (10.6)	
Grade group4	8 points		48 (22.2)	
Grade group5	≥ 9 points		79 (36.6)	
**Lymph node metastasis (%)**				
No			18 (8.3)	
Yes			198 (91.7)	
**Limb bone metastasis (%)**				
No			150 (69.4)	
Yes			66 (30.6)	
**Axial bone metastasis (%)**				
No			24 (11.1)	
Yes			192 (88.9)	
**Visceral metastasis (%)**				
No			170 (78.7)	
Yes			46 (21.3)	
**Alkaline phosphatase (U/L)**			188.71±185.14 (42-973)	
**Lactate dehydrogenase (U/L)**			223.94±185.17 (66-1392)	
**Hemoglobin (g/L)**			125.76±20.23 (58-153)	
**PSA baseline level (%)**				
≤ 65 ng/ml			168 (77.8)	
> 65 ng/ml			48 (22.2)	
**nPSA (%)**				
≤ 0.2 ng/ml			82 (38.0)	
> 0.2 ng/ml			134 (62.0)	
**TTN (%)**			8.10±6.99 (2-42)	
< 6 months			115 (53.2)	
≥ 6 months			101 (46.8)	

**Table 2:** Univariate analysis for progression to metastatic castration resistant prostate cancer.

	HR	95%CI	P Value
**Age**	1.006	0.986-1.025	0.571
**BMI**	0.981	0.931-1.035	0.487
**Gleason grade group**			
Grade group1 ≤ 6 points	1.000		
Grade group2 3+4 points	1.493	0.562-3.963	0.042*
Grade group3 4+3 points	2.680	1.226-5.857	0.013*
Grade group4 8 points	8.722	4.043-18.818	<0.001*
Grade group5 ≥ 9 points	13.181	6.218-27.942	<0.001*
P for trend <0.001*			
**Lymph node metastasis**			
No	1.000		
Yes	1.022	0.926-1.496	0.248
**Limb bone metastasis**			
No	1.000		
Yes	1.871	1.332-2.628	<0.001*
**Axial bone metastasis**			
No	1.000		
Yes	0.847	0.502-1.428	0.532
**Visceral metastasis**			
No	1.000		
Yes	0.625	0.406-0.960	0.032*
**Alkaline phosphatase**	1.001	1.000-1.002	0.019*
**Lactate dehydrogenase**	1.001	1.000-1.001	0.116
**Hemoglobin**	0.993	0.985-1.002	0.113
**PSA baseline level**			
≤ 65 ng/ml	1.000		
> 65 ng/ml	2.036	1.411-2.939	<0.001*
**nPSA**			
≤ 0.2ng/ml	1.000		
> 0.2ng/ml	6.172	3.944-9.658	<0.001*
**TTN**			
< 6 months	1.000		
≥ 6 months	0.111	0.072-0.170	<0.001*

*: P<0.05

**Table 3:** Multivariate analysis for progression to metastatic castration resistant prostate cancer.

	HR	95%CI	P Value
**Gleason grade group**			
Grade group1 ≤ 6 points	1.000		
Grade group2 3+4 points	1.512	0.556-4.108	0.418
Grade group3 4+3 points	3.169	1.403-7.160	0.006*
Grade group4 8 points	4.335	1.901-9.884	<0.001*
Grade group5 ≥ 9 points	5.159	2.312-11.512	<0.001*
P for trend <0.001*			
**Limb bone metastasis**			
No	1.000		
Yes	1.164	0.787-1.724	0.447
**Visceral metastasis**			
No	1.000		
Yes	0.737	0.419-1.297	0.290
**Alkaline phosphatase**	1.000	0.999-1.001	0.931
**PSA baseline level**			
≤ 65ng/ml	1.000		
> 65ng/ml	1.141	0.776-1.677	0.501
**nPSA**			
≤ 0.2ng/ml	1.000		
> 0.2ng/ml	2.665	1.495-4.750	0.001*
**TTN**			
< 6 months	1.000		
≥ 6 months	0.262	0.161-0.426	<0.001*

*: P<0.05
